# Isolation and Expression of a cDNA Encoding Methylmalonic Aciduria Type A Protein from *Euglena gracilis* Z

**DOI:** 10.3390/metabo3010144

**Published:** 2013-02-18

**Authors:** Yukinori Yabuta, Ryota Takamatsu, Satoshi Kasagaki, Fumio Watanabe

**Affiliations:** School of Agricultural, Biological, and Environmental Sciences, Faculty of Agriculture, Tottori University, 4-101 Koyama-Minami, Tottori 680-8553, Japan; E-Mails: yabuta@muses.tottori-u.ac.jp (Y.Y.); ascorbate250@gmail.com (R.T.); cobalaminb12@gmail.com (S.K.)

**Keywords:** cobalamin, *Euglena gracilis*, methylmalonyl-CoA mutase, *MMAA*, vitamin B_12_

## Abstract

In animals, cobalamin (Cbl) is a cofactor for methionine synthase and methylmalonyl-CoA mutase (MCM), which utilizes methylcobalamin and 5′-deoxyadenosylcobalamin (AdoCbl), respectively. The *cblA* complementation class of inborn errors of Cbl metabolism in humans is one of three known disorders that affect AdoCbl synthesis. The gene responsible for *cblA* has been identified in humans (*MMAA*) as well as its homolog (*meaB*) in *Methylobacterium extorquens*. Recently, it has been reported that human *MMAA* plays an important role in the protection and reactivation of MCM *in vitro.* However, the physiological function of *MMAA* is largely unknown. In the present study, we isolated the cDNA encoding *MMAA* from *Euglena gracilis* Z, a photosynthetic flagellate. The deduced amino acid sequence of the cDNA shows 79%, 79%, 79% and 80% similarity to human, mouse, *Danio rerio MMAAs* and *M. extorquens MeaB*, respectively. The level of the MCM transcript was higher in Cbl-deficient cultures of *E. gracilis* than in those supplemented with Cbl. In contrast, no significant differences were observed in the levels of the *MMAA* transcript under the same two conditions. No significant difference in MCM activity was observed between *Escherichia coli* that expressed either MCM together with *MMAA* or expressed MCM alone.

## 1. Introduction

Vitamin B_12_, or cobalamin (Cbl), is an organometallic cofactor that supports the activities of enzymes in organisms, ranging from bacteria to humans [1,2]. In animals, Cbl is the cofactor for cytosolic methionine synthase (EC 2.1.1.13) and mitochondrial methylmalonyl-CoA mutase (MCM, EC 5.4.99.2), more specifically, methylcobalamin (MeCbl) and 5′-deoxyadenosylcobalamin (AdoCbl), respectively. Inhibition of Cbl transport or biochemical modifications of Cbl usually causes severe diseases [3]. The *cblA* complementation class of inborn errors of Cbl metabolism is one of three known disorders that affect AdoCbl synthesis without affecting the synthesis of MeCbl [4]. The gene responsible for *cblA* has been identified through the examination of prokaryotic gene arrangements and is called *MMAA* [3]. The deduced amino acid sequences of human or mouse MMAA show homology with the periplasmic protein kinase ArgK of *Escherichia coli* [3]. ArgK is a coupled ATPase of the lysine, arginine and ornithine (LAO) transport system [5], suggesting the possibility that MMAA is a component of a transporter or an accessory protein that is involved in the translocation of Cbl into mitochondria [3].

MCM and a homolog of *MMAA* (*MeaB*) are present in the genome of the methylotrophic bacterium *Methylobacterium extorquens* AM1 [6]. This bacterium synthesizes AdoCbl and utilizes AdoCbl as the cofactor for MCM and *meaA* (ethylmalonyl-CoA mutase). The mutation in *meaB* has no effect on the function of *meaA*. However, total MCM activity was significantly reduced in *meaB* mutants compared with that of the wild-type, although the *meaB* mutants synthesized AdoCbl [7]. These findings suggest that *meaB* is not involved in the biosynthesis of AdoCbl or the transport of Cbl in *M. extorquens*.

Diol and glycerol dehydratases, Cbl-dependent enzymes, undergo concomitant irreversible inactivation by glycerol during catalysis [8]. This inactivation involves irreversible cleavage of the Co-C bond of AdoCbl, forming 5′-deoxyadenosine and an alkylcobalamin-like species. The Cbl species that is formed remains tightly bound to the enzyme, inactivating the enzyme irreversibly. Diol or glycerol dehydratase-reactivating factor participates in reactivation of the inactivated holoenzymes by mediating ATP-dependent exchange of the modified coenzyme for free intact coenzyme [9,10,11,12].

Korokova and Lidstrom [7] found that *meaB* forms a complex with MCM and stimulates MCM activity *in vitro*. However, neither cyanocobalamin (CNCbl) nor AdoCbl is released from MCM in the presence of *meaB*. Further, purified *meaB* did not restore MCM activity in the extracts prepared from the *meaB* mutant. Therefore, they proposed that *meaB* does not function as a reactivating factor, but is involved in protection of MCM from suicide inactivation by possibly providing a stabilization function. However, recently, it has been reported that *meaB* involved in AdoCbl trafficking to MCM functions as an editor, discriminating between inactive and active cofactor forms and permitting transfer only of AdoCbl, in a process that is gated by GTP hydrolysis [13]. Furthermore, the editing function of *meaB* is also used for reactivating inactivated MCM formed occasionally during turnover. Takahashi-Íñiguez *et al.* [14] have reported that human *MMAA* plays as two important roles as both the “protectase” and reactivase of MCM. In *E. coli*, sleeping beauty mutase (sbm; MCM homolog) interacts with *ygfD* (the *MMAA* or *meaB* homolog) [15]. 

*Euglena gracilis* Z, a photosynthetic flagellate, possesses characteristics of both plant and animal cells [16] and requires Cbl for its growth [17,18]. It has been reported that *E. gracilis* has Cbl-dependent methionine synthase and ribonucleotide redactase [19,20]. Furthermore, the purification of aquacobalamin reductase form *E. gracilis* was reported [21]. Recently, we reported that *E. gracilis* MCM is a homodimer and is present in mitochondria [22]. Further, we isolated a full-length *E. gracilis* MCM cDNA whose deduced amino acid sequence indicates that it is a mitochondrial protein. These findings suggest the possibility that *E. gracilis*, human and *M. extorquens* MCMs share molecular properties. To explore the function of *MMAA*, we isolated a cDNA encoding *MMAA* from *E. gracilis* and measured MCM activity in *E. coli* that expressed MCM alone or together with *MMAA.*

## 2. Results and Discussion

### 2.1. Isolation and Characterization of a cDNA Encoding *MMAA*

To isolate a cDNA encoding *E. gracilis MMAA*, a BLASTP search was performed against the *Euglena* EST data base [23] using the amino acid sequence of human *MMAA* [3] as a query. This search revealed an EST clone (cluster ID ELL00001639) encoding a putative *E. gracilis MMAA*. Similar to other *MMAA* or *meaB*, this EST clone is annotated as a putative periplasmic protein kinase ArgK. The corresponding full-length cDNA was cloned using the rapid amplification of cDNA ends method, and its sequence was submitted to the DDBJ/GenBank/EMBL data base (accession number AB772316). In *E. gracilis*, the short sequences present at the 5′ end of small cytoplasmic mRNAs [24,25,26] are transferred to pre-mature mRNAs by a *trans*-splicing mechanism. The presence of a spliced leader sequence at the 5′ end of *E. gracilis MMAA* cDNA (5′-TTTTTTTTCG-3′) indicated that full-length *MMAA* cDNA was obtained. The cDNA contains 1,400 bp with an open reading frame of 1,056 bp predicted to encode 352 amino acids residues (with a calculated molecular mass of 39113.9 Da).

**Figure 1:**
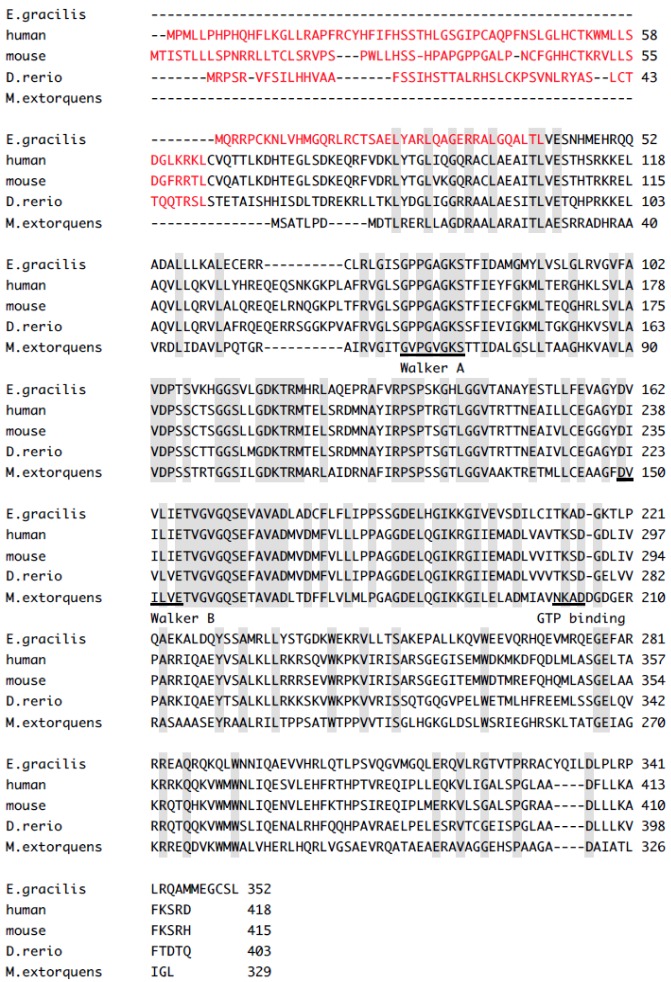
Comparison of the deduced amino acid sequences of *MMAA* from *E. gracilis*, human, mouse, *D. rerio* and *meaB* from *M. extorquens*. Sequences were aligned using ClustalW [29]. Sequence motifs defining the G3E family of proteins are indicated, including the GxxGxGK[S/T] Walker A and the DHbHbHbHbE Walker B motif. Hb denotes hydrophobic residues and an [N/T]KxD GTP-binding motif, as described by Leipe *et al.* [30], is underlined. Red letters indicate the mitochondrial leader sequence. Amino acids that are identical in all species are shaded gray.

The deduced amino acid sequence of the *E. gracilis MMAA* cDNA shows 79%, 79%, 79% and 80% similarity to human (NP_758454), mouse (NP_598584), *Danio rerio* (NP_001098582) *MMAAs* and *M. extorquens* (AAL86727) *meaB*, respectively (Figure 1). The *E. gracilis MMAA* cDNA encodes a predicted protein of 352 amino acid residues and includes Walker A and Walker B motifs, as well as a GTP-binding sequence [3]. The presence of a signal peptide and cleavage sites was predicted by the TargetP prediction program [27]. However, the cleavage site is present in the relatively well-conserved region among the *MMAAs* of vertebrates (Figure 1), suggesting the possibility that the *MMAA* cleavage site in *E. gracilis* is located closer to its N-terminal region.

### 2.2. Expression of MMAA by E. gracilis

Although it is known that the levels of MCM protein and total MCM activity in the livers of Cbl-deficient rats are higher than those in the livers of rats whose diet was supplemented with Cbl, the level of the MCM transcript in the livers of rats fed a Cbl-deficient diet was lower than that in the livers of rats fed Cbl-supplemented diets [28]. To explore the regulation of the expression of *MCM* and *MMAA*, we analyzed the levels of their respective transcripts in response to Cbl in Cbl-deficient *E. gracilis*.

**Figure 2:**
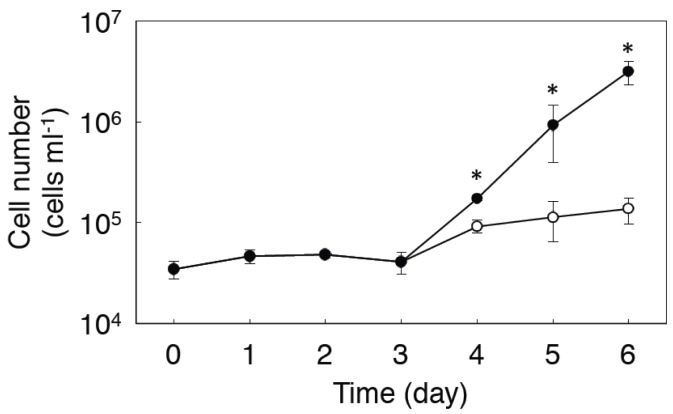
Effect of the addition of CNCbl on the growth of Cbl-deficient *E. gracilis* cells. Open circles are Cbl-deficient cells. Closed circles are Cbl-supplemented cells. The data are the mean value ± SD of three individual experiments. Asterisks indicate that the mean values are significantly different compared with those of Cbl-deficient *E. gracilis* cells (*p* < 0.05).

Cells cultured (1 mL) for one week were transferred to CNCbl-free medium, cultured for another week and then transferred (1 mL) to CNCbl-free medium. After three days, CNCbl (5 µg/mL) was added, and the cells were cultured for another three days (closed circles; Figure 2). Under the Cbl deficient condition, the growth of *E. gracilis* cells was highly suppressed (open circles; Figure 2). The addition of CNCbl increased the cell growth.

**Figure 3:**
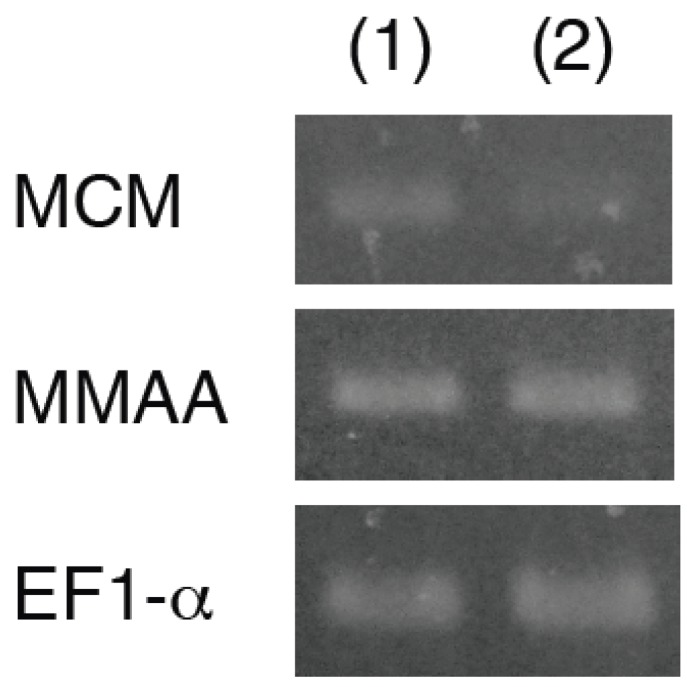
Semi-quantitative real-time (RT)-PCR analysis of the levels of *MCM* and *MMAA* transcripts in (1) Cbl-deficient and (2) Cbl-supplemented *E. gracilis* cultures. Fragments corresponding to *MCM*, *MMAA*, and *EF1-α* were amplified using PCR.

In three-day cultured Cbl-deficient *E. gracilis* cells, total MCM activity was 8.54 nmol min^−1^ mg^−1^ protein. At three days after the addition of Cbl to Cbl-deficient cells, total MCM activity was decreased to 38%, while that in the Cbl-deficient cells remained high (89%). In Cbl-deficient *E. gracilis* cells, the level of the *MCM* transcript was 2.3-fold higher than that in the Cbl-supplemented *E. gracilis* cells (Figure 3).

In contrast, no significant differences in the levels of the *MMAA* transcript were observed between the Cbl-deficient and Cbl-supplemented *E. gracilis*, suggesting the possibility that *MMAA* is constitutively expressed these cells.

### 2.3. Effect of Coexpression of *MMAA* on *MCM* Activity in E. coli

As noted above, *MCM* activity is significantly decreased in *meaB* mutants of *M. extorquens* [7]. To examine the effect of the expression of *MMAA* on *MCM* activity, the expression vectors pET/Eg.MCM and pCDF/Eg.MMAA were transformed together or individually in *E. coli* using the expression vectors described below*.* In cells transformed with either the combinations of pET-16b and pCDF/Eg.MMAA or pET-16b and pCDF-1b, *MCM* activity was below the detection limit of the assay (5 pmol min^−1^ mg^−1^ protein) (Table 1). No significant difference in *MCM* activity was detected between cells expressing *MCM* alone or together with *MMAA .* We checked *E. gracilis* MMAA protein in *E. coli* cells using anti His-tag antibody. *E. gracilis* MMAA protein was slightly expressed in the soluble fraction in the pCDF-1b/Eg.MMAA transformed *E. coli*, suggesting that the expression level of *E. gracilis* MMAA protein was very low.

**Table 1:** *MCM* activity in *E. coli* expressing *MCM* or *MMAA* together or individually.

Vector pairs	MCM activity (nmol min^−1^ mg^−1^ protein)
pET/Eg.MCM - pCDF-1b	12.1 ± 2.0
pET-16b - pCDF-1b/Eg.MMAA	< 0.005
pET-16b - pCDF-1b	< 0.005
pET/Eg.MCM - pCDF/Eg.MMAA	11.6 ± 1.8

Data are mean values ± standard deviation from three assays.

*E. coli* *sbm* and *ygfD* genes are contained in an operon comprising *sbm-ygfD-ygfG-ygfH* [31]. *E. coli sbm* forms a homodimer [14] as its *E. gracilis* homolog. Further, the deduced amino acid sequence of *E. coli sbm* is 89% similar to that of *E. gracilis MCM*. These findings suggest the possibility that *ygfD* functions to protect *E. gracilis MCM* from inactivation and acts to reactivate it in *E. coli*. Previous findings, and our presented data suggests the possibility that the function of *E. gracilis* *MMAA* was similar to those of human *MMAA* and *meaB*.

In *Caenorhabditis elegans*, the suppression of *MMAA*, *MMAB* [co(I)balamin adenosyltransferase] or *MCM* expression using RNAi technology caused the increase of methylmalonic acid [32]. To clarify the Cbl metabolism, including the function of *MMAA* in *E. gracilis*, analyses using recombinant proteins and RNAi technology are necessary.

## 3. Experimental Section

### 3.1. E. gracilis and Culture Conditions

*E. gracilis* strain *Z* was grown in Koren–Hutner medium under continuous illumination at a photosynthetic photon flux density of 24 µmol m^−2^ s^−1^ at 26 °C until the cells reached stationary phase (6 days) [33]

### 3.2. RT-PCR-Amplification and Sequence Analysis

Based on analysis of the *Euglena* EST database using the Protest EST Program [23], a gene-specific primer was designed to amplify the missing 3′ and 5′ ends of the *MMAA* EST sequence (ELL00001639). Total RNA was isolated from *E. gracilis* using Sepasol-RNA I (Nakalai Tesque, Kyoto, Japan). The primers 5′-TGGTGGAGTCAAACCACATG-3′ and 5′- TCAGACACTTCCACAATGCC -3′ were used sequentially for 3′-rapid amplification of cDNA ends (3′-RACE) or 5′-RACE with a GeneRacer^TM^ Kit (Life Technologies, Carlsbad, CA, USA). Amplified fragments were cloned into pSTBlue-1, and the nucleotide sequences of the 3′- or 5′-extended fragments were determined using an ABI Prism 3100 Genetic Analyzer (Applied Biosystems, Foster City, CA, USA). The full-length coding sequence of *Euglena MMAA* was amplified using PCR primed by Eg.MMAA-F (5′-ATTCCATGCAACGCCGCCCA-3′) and Eg.MMAA-R (5′-CCACGTATGCTTTCCTCCTT-3′). PCR amplification was carried out using PrimeSTAR (Takara Shuzo, Kyoto, Japan). A single-stranded cDNA prepared from 3-day *Euglena* cultures was used as template. The amplified product was cloned into pZErO-2 (Life Technologies), and the sequence was verified using an ABI Prism 3100 Genetic Analyzer (Applied Biosystems).

### 3.3. Semi-Quantitative RT-PCR Analysis

Semi*-*quantitative RT-PCR analysis was performed according to Ishikawa *et al.* [22]. Primer pairs were as follows: EF1-α-F (5′-GTTGACCCTCATTGGTGCTT-3′); EF1-α-R (5′-CTTGGTCACCTTCCCAGTGT-3′); Eg.MCM-F (5′-GTCCATCGACAACACCATTG -3′); Eg.MCM-R (5′-CGACTTCAGCTCGTTGATGA -3′); and Eg.MMAA-F (5′-ATTCCATGCAACGCCGCCCA-3′); Eg.MMAA-R (5′-TCAGACACTTCCACAATGCC-3′). The experiments were repeated at least three times with cDNA prepared from three batches of *E. gracilis* cultures. The quantitative intensity was determined by applying densitometry to images of the blots [34].

### 3.4. Construction of *MMAA* and *MCM* Expression Plasmids

To construct *MMAA* and *MCM* expression vectors, their open reading frames were amplified from the first strand cDNAs, using the primer sets as follows: MMAA-*Pma* CI-F (5′-TCACCACCACCATCACGTGATGCAACGCCGCCCATGCAA-3′), MMAA-*Pma* CI-R (5′-TCGAACCGGTACCCACGTGCTACAGGCTGCAGCCCTCCA-3′), MCM-*Nde* I-F (5′-CATATGATCGACCTTCCACCAAAGTG-3′) and MCM-*Bam* HI-F(5′-GGATCCTCAGAGTTTGTTCAGGACCT-3′). The amplified DNA fragments were ligated into the vector pZErO-2 (Life Technologies), and the sequence was verified using an ABI Prism 3100 Genetic Analyzer (Applied Biosystems). The resulting constructs were digested with *Pma* CI for *MMAA* or *Nde* I/*Bam* HI for *MCM* and ligated into the expression vectors pCDF-1b (Novagen, Madison, WI) or pET16b (Novagen) to produce histidine-tagged proteins. The resulting constructs were designated pCDF/Eg.MMAA and pET/Eg.MCM, respectively.

### 3.5. Expression of Recombinant MMAA or MCM Proteins

To explore the effect of the expression of Eg.MMAA on MCA activity in *E. coli*, four plasmids sets (pET16-b and pCDF-1b, pET/Eg.MCM and pCDF-1b, pET16-b and pCDF/Eg.MMAA and pET/Eg.MCM and pCDF/Eg.MMAA) were introduced into *E. coli* strain BL21 Star (DE3) (Life Technologies). Transformed *E. coli* were cultured in 50 mL LB medium supplemented with ampicillin (50 µg mL^−1^) and chloramphenicol (34 µg mL^−1^) at 37 °C overnight. The culture was then transferred to Luria–Bertani medium (1 l). When the culture reached an absorbance of 0.6 at 600 nm, 0.4 mM isopropyl β-D-thiogalactopyranoside was added, and the bacteria were grown for another 6 h at 37 °C. The cells were harvested by centrifugation at 6,000 × *g* for 10 min, and the pellets were kept frozen at −20 °C.

### 3.6. Enzyme Extraction and Assay

*E. coli* pellets were resuspended in 100 mM Tris-HCl, pH 7.5, sonicated (Tomy, Tokyo, Japan) (10 kHz) using 20 s strokes with 30 s intervals and centrifuged at 15,000 × g for 15 min. *MCM* activity was assayed by a modified HPLC method described by Gaire *et al.* (1999). Briefly, the assay mixture (0.15 mL) for determining total *MCM* activity contained 100 mM potassium phosphate buffer (pH 7.5), 30 µM AdoCbl, 0.15 mM (*R, S*)-methylmalonyl-CoA and enzyme. The components, except for (*R, S*)-methylmalonyl-CoA, were mixed in microcentrifuge tubes in the dark, and the temperature was equilibrated by incubation in a heating bucket (e-Heating Bucket EHB, TAITEC Cop., Saitama, Japan) maintained at 3 °C. The reaction mixture was preincubated for 5 min, and the reaction was started by the addition of (*R, S*)-methylmalonyl-CoA and maintained for 5 min. The enzyme reaction was stopped by the addition of 50 µL of 10% (w/v) trichloroacetic acid. The reaction mixture was filtered through a 0.45 µM membrane filter (Millex Syringe Driven Filter Unit, LH-type, Milli-pore, USA). An aliquot (20 µL) of the filtrate was analyzed using a Shimadzu HPLC apparatus (two LC-10ADvp pumps, DGV-12A degasser, SCL-10Avp system controller, SPD-10Avvp ultraviolet-visible detector, CTO-10Avp column oven, 100 µL sample loop and C-R6A Chromatopac Integrator). The sample (20 µL) was loaded onto a reversed-phase HPLC column (Cosmosil 5C18-AR-II, Φ3.0 × 150 mm) equilibrated with 50% solvent A (100 mM acetic acid in 100 mM potassium phosphate buffer, pH 7.0) and 50% solvent B [18% (v/v) methanol in solvent A]. (*R, S*)-methylmalonyl-CoA and succinyl-CoA were eluted with a linear gradient of methanol (50%–100% solvent B) for 7.0 min at 40 °C and monitored by measuring the absorbance at 254 nm. The flow rate was 1.0 mL min^−1^. *MCM* activity was calculated from the amount of succinyl-CoA formed. Protein concentrations were determination using a Quant-iT™ Protein Assay and a Qubit fluorometer (Life Technologies).

## 4. Conclusion

*E. gracilis MMAA* cDNA contained an open reading frame encoding the protein of 352 amino acids with a calculated molecular mass of 39113.9 Da, preceded by a putative mitochondrial targeting signal consisting of 41 amino acid residues. No significant differences in the levels of the *MMAA* transcript were observed between the Cbl-deficient and Cbl-supplemented *E. gracilis*. No significant difference in MCM activity was observed between *E. coli* that expressed either MCM together with MMAA or expressed MCM alone. Previous findings, and our presented data suggests the possibility that the function of *E. gracilis* *MMAA* was similar to those of human *MMAA* and *meaB*.

## References

[1] Banerjee R., Ragsdale S.W. (2003). The many faces of vitamin B_12_: Catalysis by cobalamin-dependent enzymes. Annu. Rev. Biochem..

[2] Ueta K., Ishihara Y., Yabuta Y., Masuda S., Watanabe F. (2011). TLC-analysis of a corrinoid compound from Japanese rock-oyster “Iwa-gaki” (*Crassostrea. nippona*). J. Liquid Chromatography Related Technol..

[3] Dobson C.M., Wai T., Leclerc D., Wilson A., Wu X., Doré C., Hudson T., Rosenblatt D.S., Gravel R.A. (2002). Identification of the gene responsible for the cblA complementation group of vitamin B_12_-responsive methylmalonic acidemia based on analysis of prokaryotic gene arrangements. Proc. Natl. Acad. Sci. USA.

[4] Lerner-Ellis J.P., Dobson C.M., Wai T., Watkins D., Tirone J.C., Leclerc D., Doré C., Lepage P., Gravel R.A., Rosenblatt D.S. (2004). Mutations in the MMAA gene in patients with the cblA disorder of vitamin B_12_ metabolism. Hum. Mutat..

[5] Celis R.T., Leadlay P.F., Roy I., Hansen A. (1998). Phosphorylation of the periplasmic binding protein in two transport systems for arginine incorporation in *Escherichia coli* K-12 is unrelated to the function of the transport system. J. Bacteriol..

[6] Korotkova N., Chistoserdova L., Kuksa V., Lidstrom M.E. (2002). Glyoxylate regeneration pathway in the methylotroph *Methylobacterium. extorquens* AM1. J. Bacteriol..

[7] Korotkova N., Lidstrom M.E. (2004). MeaB is a component of the methylmalonyl-CoA mutase complex required for protection of the enzyme from inactivation. J. Biol. Chem..

[8] Toraya T. (2000). Radical catalysis of B_12_ enzymes: structure, mechanism, inactivation, and reactivation of diol and glycerol dehydratases. Cell. Mol. Life Sci..

[9] Mori K., Tobimatsu T., Toraya T. (1997). A protein factor is essential for *in situ* reactivation of glycerol-inactivated adenosylcobalamin-dependent diol dehydratase. Biosci. Biotechnol. Biochem..

[10] Mori K., Tobimatsu T., Hara T., Toraya T. (1997). Characterization, sequencing, and expression of the genes encoding a reactivating factor for glycerol-inactivated adenosylcobalamin-dependent diol dehydratase. J. Biol. Chem..

[11] Toraya T., Mori K. (1999). A reactivating factor for coenzyme B_12_-dependent diol dehydratase. J. Biol. Chem..

[12] Kajiura H., Mori K., Tobimatsu T., Toraya T. (2001). Characterization and mechanism of action of a reactivating factor for adenosylcobalamin-dependent glycerol dehydratase. J. Biol. Chem..

[13] Padovani D., Banerjee R. (2009). A G-protein editor gates coenzyme B_12_ loading and is corrupted in methylmalonic aciduria. Proc. Natl. Acad. Sci. USA.

[14] Takahashi-Íñiguez T., García-Arellano H., Trujillo-Roldán M.A., Flores M.E. (2011). Protection and reactivation of human methylmalonyl-CoA mutase by MMAA protein. Biochem. Biophys. Res. Commun..

[15] Froese D.S., Dobson C.M., White A.P., Wu X., Padovani D., Banerjee R., Haller T., Gerlt J.A., Surette M.G., Gravel R.A. (2009). Sleeping beauty mutase (sbm) is expressed and interacts with ygfd in *Escherichia coli*. Microbiol. Res..

[16] Kitaoka S., Nakano Y., Miyatake K., Yokota A., Buetow D.E. (1989). In The Biology of Euglena.

[17] Isegawa Y., Nakano Y., Kitaoka S. (1984). Conversion and distribution of cobalamin in *Euglena gracilis* Z, with special reference to its location and probable function within chloroplasts. Plant Physiol..

[18] Watanabe F., Nakano Y., Stupperich E. (1992). Different corrinoid specificities for cell growth and the cobalamin uptake system in *Euglena gracilis* Z. J. Gen. Microbiol..

[19] Isegawa Y., Watanabe F., Kitaoka S., Nakano Y. (1994). SubcelluIar distribution of cobalamin-dependent methionine synthase in *Euglena*
*gracilis* z. Phytochemisty.

[20] Torrents E., Trevisiol C., Rotte C., Hellman U., Martin W., Reichard P. (2006). *Euglena gracilis* ribonucleotide reductase: the eukaryote class II enzyme and the possible antiquity of eukaryote B_12_ dependence. J. Biol. Chem..

[21] Watanabe F., Oki Y., Nakano Y., Kitaoka S. (1987). Purification and characterization of aquacobalamin reductase (NADPH) from *Euglena gracilis*. J. Biol. Chem..

[22] Miyamoto E., Tanioka Y., Nishizawa-Yokoi A., Yabuta Y., Ohnishi K., Misono H., Shigeoka S., Nakano Y., Watanabe F. (2010). Characterization of methylmalonyl-CoA mutase involved in the propionate photoassimilation of *Euglena gracilis* Z. Arch. Microbiol..

[23] Taxonomically Broad EST Database. http://amoebidia.bcm.umontreal.ca/pepdb/searches/login.php.

[24] Tessier L.H., Keller M., Chan R.L., Fournier R., Weil J.H., Imbault P. (1991). Short leader sequences may be transferred from small RNAs to pre-mature mRNA by trans-splicing in *Euglena*. EMBO J..

[25] Frantz C., Ebel C., Paulus F., Imbaut P. (2000). Characterization of *trans*-splicing in Euglenoids. Curr. Genet..

[26] Ishikawa T., Tajima N., Nishikawa H., Gao Y., Rapolu M., Shibata H., Sawa Y., Shigeoka S. (2010). *Euglena gracilis* ascorbate peroxidase forms an intramolecular dimeric structure: its unique molecular characterization. Biochem. J..

[27] TargetP prediction program. http://www.cbs.dtu.dk/services/TargetP/.

[28] Nakao M., Hironaka S., Harada N., Adachi T., Bito T., Yabuta Y., Watanabe F., Miura T., Yamaji R., Inui H., Nakano Y. (2009). Cobalamin deficiency results in an abnormal increase in L-methylmalonyl-co-enzyme-A mutase expression in rat liver and COS-7 cells. Br. J. Nutr..

[29] ClustalW Program. http://clustalw.ddbj.nig.ac.jp/index.php?lang=ja,.

[30] Leipe D., Wolf Y., Koonin E., Aravind L. (2002). Classification and evolution of P-loop GTPases and related ATPases. J. Mol. Biol..

[31] Haller T., Buckel T., Rétey J., Gerlt J.A. (2000). Discovering new enzymes and metabolic pathways: conversion of succinate to propionate by *Escherichia coli*. Biochemistry.

[32] Chandler R.J., Aswani V., Tsai M.S., Falk M., Wehrli N., Stabler S., Allen R., Sedensky M., Kazazian H.H., Venditti C.P. (2006). Propionyl-CoA and adenosylcobalamin metabolism in *Caenorhabditis. elegans*: Evidence for a role of methylmalonyl-CoA epimerase in intermediary metabolism. metabolism. Mol. Genet. MeTable.

[33] Koren L.E., Hutner S.H. (1967). High-yield media for photosynthesizing *Euglena gracilis* Z. J. Protozool..

[34] Image J. http://rsbweb.nih.gov/ij/,.

[35] Enago. http://www.enago.jp/,.

